# Trends and correlates of HIV prevalence among adolescents in South Africa: evidence from the 2008, 2012 and 2017 South African National HIV Prevalence, Incidence and Behaviour surveys

**DOI:** 10.1186/s12981-021-00422-3

**Published:** 2021-12-14

**Authors:** Musawenkosi Mabaso, Goitseone Maseko, Ronel Sewpaul, Inbarani Naidoo, Sean Jooste, Sinovuyo Takatshana, Tarylee Reddy, Khangelani Zuma, Nompumelelo Zungu

**Affiliations:** 1 Human and Social Capabilities Research Division, Human Sciences Research Council, Pretoria, South Africa; 2 Biostatistics Unit, South African Medical Research Council, Durban, South Africa; 3 Department of Psychology, University of Pretoria, Pretoria, South Africa

**Keywords:** Trends in HIV prevalence, Adolescents, Risk factors, South Africa

## Abstract

**Background:**

Adolescents are at increased risk of HIV infection compared to other age groups. There is an urgent need for strategic information that will inform programmes to reduce risk and vulnerability to HIV and reverse the pattern of increasing HIV infection as they transition to adulthood. This paper analysed trends and factors associated with HIV prevalence among adolescents in South Africa using the national HIV population-based household surveys conducted in 2008, 2012 and 2017.

**Methods:**

All three surveys used a multistage cross-sectional design. A trend analysis was conducted to assess the differences in HIV prevalence and covariates overtime using P-trend Chi-squared statistic. Univariate and multivariate logistic regression models were used to determine factors associated with HIV prevalence.

**Results:**

Overall there was a significant increase in HIV prevalence among adolescents aged 12–19 years from 3.0% (n = 2892) in 2008 to 3.2% (n = 4829) in 2012 and 4.1% (n = 3937) in 2017 (p = 0.031). The odds of being HIV positive among adolescents aged 12–19 years was significantly higher among females [AOR = 2.24; 95% CI (1.73–2.91); p < 0.001] than males, those residing in KwaZulu-Natal province [AOR = 2.01; 95% CI (1.-3.99); p = 0.027] than Northern Cape, and those who did not attend an educational institution and were unemployed [AOR = 2.66; 95% CI (1.91–3.67); p < 0.001] compared to those attending an educational institution. The odds were significantly lower among Whites [AOR = 0.29; 95% CI (0.09–0.93); p = 0.037], Coloureds [AOR = 0.21; 95% CI (0.11–0.37); p ≤ 0.001] and Indian/Asian [AOR = 0.08; 95% CI (0.02–0.34); p = 0.001] population groups than Black Africans.

**Conclusion:**

The observed increasing trend and gender disparities in HIV prevalence suggests an urgent need for age appropriate and gender specific HIV interventions tailored and targeted at identified drivers of HIV infection among adolescents.

## Introduction

Adolescents represent a growing number of people living with HIV worldwide. In 2019, about 1.7 million (1.1 million-2.4 million) adolescents between the ages of 10 and 19 were living with HIV worldwide [1]. In addition, 170,000 (53,000–340,000) adolescents between the ages of 10 and 19 were newly infected with HIV in 2019. In sub-Saharan Africa that year, four times as many adolescent girls were newly infected with HIV than adolescent boys (UNICEF, 2020) [1]. Adolescents are highly vulnerable to HIV acquisition than adult because of the transition stage of their development and the need to adapt to the rapid biological, physical and structural changes in their lives [2].

In generalised epidemics, many young adolescents living with HIV acquired the infection perinatally (during pregnancy, birth or breastfeeding) where mothers were not enrolled in prevention of mother-to-child transmission (PMTCT) programmes [2, 3]. The main mode of HIV transmission among adolescents who were not perinatally infected is unprotected heterosexual sex [4]. High-risk behaviours such as early sexual debut, inconsistent condom use, substance use (alcohol and drug use, peer pressure), and sensation-seeking behaviours have been associated with increasing HIV burden in this age group [3–5].

Furthermore, age disparate sexual relationships increase risk of HIV acquisition among adolescent girls since older men are more likely to be HIV positive, and such relationships are characterized by unprotected and coercive sex [6–8]. This is more likely among adolescent girls who often engage in such relationships for economic and other material reasons. Social norms that sustain gender-based violence are also closely linked to HIV risk among adolescent girls [9, 10]. In addition, the proportion of adolescents who have comprehensive and accurate knowledge about HIV transmission and prevention remains inadequate [4]. Low risk perception for acquisition of HIV infection is also a factor in this age cohort. Another challenge is the limited ability of adolescents to independently access HIV testing and counselling services as they face age- and gender-related restrictions [11–13]. Consequently, the lack of awareness of HIV status among adolescents living with HIV is high [13].

Evidence of the high burden of HIV among adolescents underscores the need to comprehensively assess HIV prevalence and associated factors in this age group in order to generate evidence to drive policy and action. Intervening during early adolescence can shape attitudes and behaviours as they are being formed, rather than attempting to change established behaviours during later adolescence and adulthood. This paper analysed trends and factors associated with HIV prevalence among adolescents in South Africa using national HIV population-based household surveys conducted in 2008, 2012 and 2017.

## Methods

### Survey design and data collection

This secondary analysis is based on data collected using a multi-stage cross-sectional design from the three nationally representative household-based South African National HIV Prevalence, Incidence, Behaviour and Communication surveys completed in South Africa since 2008, 2012 and 2017 [14–16]. In each survey wave a systematic probability sample of 15 households was randomly chosen from 1000 enumeration areas (EAs) in 2008 and 2012 and small area layers (SALs) in 2017, which were randomly selected from 86 000 EAs based on the national sampling frame released by Statistics South Africa in 2001 and updated in 2011 [17, 18]. The selection of EAs and SALs were stratified by province and locality types namely urban formal, urban informal, rural formal (including commercial farms) and rural informal localities. In 2008 and 2012 these four locality types were used, whereas in 2017 three locality types were used as the urban informal and formal areas were grouped into one urban locality.

In the 2008 surveys, in each household a maximum of three people were selected randomly to participate in the study, each representing the 2–14 years, 15–24 years and 25 years and older age groups and three different questionnaires were administered to each age group. In the 2012 and 2017 surveys, all household members irrespective of the age were eligible to participate in the survey. In all the surveys age-appropriate questionnaires were administered to solicit information on socio-demographic characteristics, sexual practices and behaviour, knowledge, attitudes and perceptions, self-reported testing of tuberculosis and HIV, exposure to behaviour change communication campaigns, alcohol and substance use and general health related characteristics.

Dried blood spots’ (DBS) specimens were collected from consenting individuals for HIV testing. Samples were tested for HIV using an enzyme immunoassay (EIA) (Vironostika HIV Uni-Form II plus O, Biomeriux, Boxtel, The Netherlands), and samples which tested positive were retested using a second EIA (Advia Centaur XP, Siemens Medical Solutions Diagnostics, Tarrytown, New York, USA). Any samples with discordant results on the first two EIAs were tested with a third EIA (Roche Elecys 2010 HIV Combi, Roche Diagnostics, Mannheim, Germany).

### Measures

The primary outcome measure in this analysis was HIV serostatus dichotomized to a binary outcome (i.e., HIV positive = 1 and HIV negative = 0). Explanatory variables included demographic variables such as two adolescent age groups (12–14 years, 15–19 years), sex (male, female), population group as defined in the South African census (Black Africans, White, Coloured, Indian/Asian), current marital status (not married, married), school attendance (yes, no), education and employment (attend an educational institution, do not attend educational institution and unemployed, do not attend educational institution and employed), locality type (urban areas, rural areas) and province (Western Cape, Eastern Cape, Northern Cape, Free State, KwaZulu-Natal, North West, Gauteng, Mpumalanga, Limpopo), orphanhood status (yes, no). Including HIV related behavioural factors such as ever had sexual intercourse (yes, no), correct knowledge of HIV and rejection of myths (yes, no).

### Statistical analysis

All data processing and statistical analyses were done using Stata statistical software, Release 15.0 (College Station, TX: Stata Corporation). Each of the surveys had their own calculated survey weights and the methods used to derive each of these original weights are reported elsewhere [14–16]. For this analysis, the selected variables, original weights and primary sampling units (PSUs) were extracted from each survey wave and merged into a pooled dataset. A calculated relative probability weight was then derived by dividing the original survey weight by the total South African population counts in each survey year.

A chi-square statistic for trend was calculated using p-trend with the weighted estimates as inputs in order to determine changes in HIV prevalence across the study years. The analyses was further stratified by 12–14 and 15–19 year old age groups. Univariate logistic regression analysis was used to assess the associations between HIV status and selected covariates for the 12–19 year olds as a whole. Statistically significant variables were entered into a multivariate logistic regression model for the 12–19 year olds. Crude and adjusted odds ratios (aORs) with 95% confidence Intervals (CIs) were calculated. A p ≤ 0.05 was used to indicate statistical significance. The “svy” command was used to take into account survey weights for the complex multi-level sampling design.

## Results

### Trends in HIV prevalence

Table 1 shows an increase in HIV prevalence from 2008 to 2012 among adolescents aged 12–19 years. This change over time was marked among 2–14 year old adolescents as the HIV prevalence increased from 1.1% in 2008 to 3.2% in 2012 and decreased to 2.4% in 2017 (p = 0.044). In the older adolescent age group of 15–19 year olds, HIV prevalence decreased from 4.4% in 2008 to 3.2% in 2012 and increased to 6.5% in 2017.

**Table 1:** HIV Prevalence by demographic characteristics among adolescents aged 12–19 years from the 2008, 2012 and 2017 surveys, South Africa

Variables	2008		2012		2017		
	N (%)	95% CI	N (%)	95% CI	N (%)	95% CI	p-value
Total	2892 (3.0)	2.1–4.2	4829 (3.2)	2.5–4.0	3937 (4.1)	3.3–5.0	0.031
Age group							
12–14	964 (1.1)	0.5–2.4	1712 (3.2)	2.0–5.0	1564 (2.4)	1.6–3.4	0.044
15–19	1928 (4.4)	3.0–6.5	3117 (3.2)	2.4–4.1	2373 (6.5)	4.1–6.7	0.103
Sex							
Male	1391 (1.8)	0.7–4.5	2377 (1.6)	0.9–2.7	1869 (4.5)	2.4–4.9	< 0.001
Female	1501 (4.2)	3.2–5.7	2452 (4.7)	3.7–6.1	2068 (5.7)	3.7–5.9	0.816
Race							
Black African	1785 (3.6)	2.5–5.1	3400 (3.8)	3.0–4.7	2915 (5.1)	3.7–5.6	0.361
White	232 (0.1)	0.0–0.4	183 (0.0)	–	101 (0.4)	0.8–11.6	0.047
Coloured	642 (0.6)	0.1–3.1	919 (0.3)	0.1–1.2	740 (3.1)	0.3–1.5	0.474
Indian	230 (0.0)	0.0–0.1	322 (0.1)	0.0–0.7	181 (0.1)	–	0.535
Locality							
Urban areas	2023 (3.2)	1.9–5.3	2886 (2.5)	1.7–3.6	2275 (5.3)	3.1–5.4	0.058
Rural areas	869 (2.7)	1.8–4.0	1943 (3.9)	2.9–5.2	1662 (4.0)	3.1–5.4	0.600
Province							
Western Cape	434 (0.6)	0.2–2.0	489 (0.9)	0.3–2.8	444 (2.0)	1.4–5.5	0.083
Eastern Cape	418 (3.5)	1.9–6.6	736 (1.9)	1.0–3.7	445 (6.6)	2.7–8.3	0.423
Northern Cape	214 (1.2)	0.4–3.2	392 (2.1)	0.6–7.0	311 (3.2)	0.5–3.4	0.642
Free State	178 (2.3)	0.4–12.7	287 (2.0)	1.0–4.2	310 (12)	2.3–7.0	0.028
KwaZulu-Natal	495 (2.8)	1.7–4.8	1165 (5.8)	4.1–8.1	813 (4.8)	3.0–6.7	0.644
North West	229 (2.1)	0.7–6.3	331 (3.5)	1.7–7.3	343 (6.3)	1.4–5.3	0.495
Gauteng	431 (4.4)	1.6–11.2	481 (2.6)	1.3–5.3	489 (11)	2.7–7.9	0.785
Mpumalanga	221 (4.4)	2.5–7.4	456 (4.3)	2.4–7.5	386 (7.4)	3.8–10.6	0.842
Limpopo	272 (2.5)	1.1–5.9	492 (2.4)	1.1–5.3	396 (5.9)	1.3–4.1	0.977
Orphanhood status							
Yes	633 (5.1)	3.4–7.7	1071 (5.9)	3.8–8.9	863 (7.7)	6.3–11.5	0.435
No	1971 (2.2)	1.2–4.0	2778 (1.8)	1.3–2.6	2718 (4.0)	1.9–3.5	0.025
Education attendance and employment status							
Attend an educational institution	2274 (2.4)	1.5–3.9	4193 (2.8)	2.1–3.6	3256 (3.9)	2.9–4.5	0.060
Do not attend educational institution and unemployed	259 (9.0)	5.6–14.1	446 (8.5)	5.3–13.3	361 (14)	6.7–15.8	0.713
Do not attend educational institution and Employed	110 (2.3)	0.7–7.2	121 (2.5)	0.9–6.7	60 (7.2)	0.0–1.6	0.903

Among females, there was an increase in HIV prevalence from 4.2% in 2008, to 4.7% in 2012 and 5.7% in 2017, although the change was not statistically significant. A statistically significant change in HIV prevalence was observed among males over time (p < 0.001). The HIV prevalence for male adolescents declined slightly from 1.8% in 2008 to 1.6% in 2012 and then increased markedly to 4.5% in 2017.

All population groups showed an increase in HIV prevalence across the years. However, this was only statistically significant for the White population. There was heterogeneity in HIV prevalence by locality type although not statistically significant. HIV prevalence peaked in 2017 for both locality types and there was an increasing trend over time in rural areas. There was a non-significant increase in HIV prevalence among orphans from 5.1% in 2008 to 5.9% in 2012 and 7.7% in 2017, compared to a significant increase among non-orphans, peaking in 2017 (4%, p = 0.025). Similarly, there was a non-significant increase in HIV prevalence among those who attended an educational institution with 2.4% in 2008, 2.8% in 2012 and 3.9% in 2017.

Table 2 shows trends in HIV prevalence by behavioural characteristics among adolescents aged 12–14 years. There were differences in HIV prevalence over time among those who attended school with a peak in 2012 (3.2%) (p = 0.005). HIV prevalence among those who did not attend school was largely unchanged at 6.8% in 2008 and 6.1% in 2012, but it must be highlighted that the counts for these were ≤ 20. There was an increase in HIV prevalence over time among those who were sexually active but the change was not significant. Notably there was a significant change in HIV prevalence over time (p = 0.008) among 12–14 year olds who said they never had sexual intercourse. The HIV prevalence among those who said they never had sexual intercourse was approximately 1% in 2008, compared to 2.8% in 2012 and 2.5% in 2017. Although non-significant, HIV prevalence among those who had correct knowledge of HIV and rejection of myths increased from 1.8% in 2008, 2.8% in 2012 and 3.5% in 2017.

**Table 2:** HIV Prevalence by behavioural characteristics among adolescents aged 12–14 years from the 2008, 2012 and 2017 surveys, South Africa

Variables	2008			2012			2017			p-value
	%	95% CI	N	%	95% CI	N	%	95% CI	N	
School attendance										
Yes	1.0	0.4–2.4	897	3.2	2.0–5.0	1686	2.5	1.7–3.7	1447	0.005
No	6.8	1.5–26.2	16	6.1	1.9–18.1	21	0.0	–	20	< 0.001
Ever had sexual intercourse										
Yes	5.3	0.7–30.4	18	8.6	1.5–35.8	30	0.0	–	13	0.697
No	0.9	0.3–2.3	891	2.8	1.7–4.5	1650	2.5	1.7–3.7	1429	0.008
Correct knowledge and rejection of myths										
No	0.9	0.3–2.6	697	3.2	2.0–5.1	1391	2.2	1.5–3.4	1178	0.076
Yes	1.8	0.6–5.9	216	2.8	0.5–13.5	303	3.5	1.5–8.1	289	0.253

Table 3 shows trends in HIV prevalence by behavioural characteristics among adolescents aged 15–19 years. There was a significant change in HIV prevalence over time (p = 0.005) among 15–19 year olds who said they never had sexual intercourse. There was some variability in this with 3.4% in 2008 and 1.6% in 2012 compared to a peak of 4.5% in 2017. HIV prevalence among those who had correct knowledge of HIV and rejection of myths increased from 1.9% in 2008, 2.8% in 2012 and 4.5% in 2017, although not statistically significant. Furthermore, there was a significant difference in HIV prevalence over time (p = 0.006) among those who did have correct knowledge of HIV and rejection of myths with 1.9% in 2008, 2.8% in 2012 and 4.5% in 2017.

**Table 3:** HIV Prevalence by behavioural characteristics among adolescents aged 15–19 years from the 2008, 2012 and 2017 surveys, South Africa

Variables	2008		2012		2017		p-value
	N (%)	95% CI	N (%)	95% CI	N (%)	95% CI	
Employment status							
Employed	110 (2.3)	0.7–7.2	134 (2.5)	1.0–6.4	77 (0.1)	0.0–1.0	0.724
Unemployed	243 (9.2)	5.6–14.8	453 (8.1)	5.0–12.8	563 (8.3)	5.6–12.2	0.346
Student	1377 (3.8)	2.1–6.6	2412 (2.5)	1.8–3.5	1567 (4.5)	3.3–6.1	0.090
Education attendance and employment status							
Attend an educational institution	1377 (3.8)	2.1–6.6	2507 (2.5)	1.8–3.5	1809 (4.5)	3.4–5.9	0.070
Do not attend educational institution and unemployed	243 (9.2)	5.6–14.8	425 (8.5)	5.3–13.5	341 (10.9)	7.0–16.5	0.993
Do not attend educational institution and Employed	110 (2.3)	0.7–7.2	121 (2.5)	0.9–6.7	60 (0.2)	0.0–1.6	0.903
Ever had sexual intercourse							
Yes	668 (6.2)	4.3–8.9	1094 (5.7)	4.1–7.8	794 (6.7)	4.7–9.6	0.973
No	1050 (3.4)	1.5–7.7	1951 (1.6)	1.0–2.7	1377 (4.5)	3.1–6.4	0.005
Correct knowledge and rejection of myths							
No	1167 (5.7)	3.6–8.8	2266 (3.3)	2.4–4.5	1441 (5.7)	4.3–7.4	0.867
Yes	584 (1.9)	0.8–4.5	833 (2.8)	1.5–5.0	766 (4.5)	2.9–6.8	0.006

### Factors associated with HIV prevalence

Table 4 shows the results of the logistic regression analysis of factors associated with HIV prevalence among adolescents aged 12–19 years. The final multivariate regression model shows that the odds of being HIV positive were significantly more likely among females than males [aOR = 2.24 (95% CI: 1.73–2.91); p < 0.001], those residing in KwaZulu-Natal than Northern Cape province [aOR = 2.01 (95% CI: 1.09–3.99); p = 0.027], and those who did not attend an educational institution and were unemployed [aOR = 2.66 (95% CI: 0.91–3.67), p < 0.001] compared to those that attended an educational institution. Compared to the Black African population group, the odds of being HIV positive were significantly less likely for all other race groups. For Whites the aOR was 0.29 (95% CI: 0.09–0.93, p = 0.037), for Coloureds the aOR was 0.21 (95% CI: 0.11–1.37, p ≤ 0.001) and for Indians/Asians the aOR was 0.82% (95% CI: 0.02–0.34, p = 0.001). Furthermore, non-orphans were less likely to be HIV positive compared to orphans [aOR = 0.45 (0.36–0.58), p < 0.001). Those who said they never had sex were less likely to be HIV positive compared to those who said they ever had sex [aOR = 0.55 (0.41–0.74), p < 0.001].

**Table 4:** Logistic regression models of factors associated with HIV prevalence among adolescents aged 12–19 years

Variables	Univariate regression			Multivariate regression		
	OR	95% CI	p-value	AOR	95% CI	p-value
Year						
2008	Ref	–	–	Ref	–	–
2012	1.00	0.76–1.32	0.997	0.98	0.70–1.37	0.927
2017	1.31	1.00–1.73	0.051	1.34	0.99–1.83	0.061
Age in years						
12–14	Ref	–	–	Ref	–	–
15–19	1.80	1.42–2.30	< 0.001	1.14	0.84–1.55	0.387
Sex						
Male	Ref	–	–	Ref	–	–
Female	2.59	2.05–3.27	< 0.001	2.24	1.73–2.91	< 0.001
Race						
African	Ref	–	–	Ref	–	–
White	0.18	0.07–0.48	0.001	0.29	0.09–0.93	0.037
Coloured	0.17	0.10–0.28	< 0.001	0.21	0.11–0.37	< 0.001
Indian/Asian	0.09	0.03–0.29	< 0.001	0.08	0.02–0.34	0.001
Locality						
Rural areas	Ref	–	–	Re	–	–
Urban areas	0.60	0.48–0.73	< 0.001	1.14	0.73–1.24	0.685
Province						
Northern Cape	Ref	–	–	Ref	–	–
Western Cape	0.81	0.40–1.61	0.541	1.42	0.66–3.06	0.368
Eastern Cape	1.95	1.09–3.49	0.025	1.49	0.76–2.95	0.248
Free State	2.10	1.10–3.99	0.024	1.61	0.78–3.33	0.193
KwaZulu-Natal	2.71	1.57–4.67	< 0.001	2.01	1.09–3.99	0.027
North West	1.79	0.94–3.40	0.075	0.99	0.47–2.08	0.982
Gauteng	1.73	0.95–3.16	0.074	1.31	0.65–2.62	0.447
Mpumalanga	3.48	1.96–6.19	< 0.001	1.93	0.97–3.82	0.059
Limpopo	1.44	0.76–2.73	0.261	0.70	0.32–1.54	0.375
Education attendance and employment status						
Attend an educational institution	Ref	–	–	Ref	–	–
Do not attend educational institution and unemployed	3.48	2.71–4.47	< 0.001	2.66	1.91–3.67	< 0.001
Do not attend educational institution and Employed	1.36	0.71–2.59	0.349	1.07	0.38–3.01	0.894
Orphanhood status						
Yes	Ref	–	–	Ref	–	–
No	0.34	0.27–0.43	< 0.001	0.45	0.36–0.58	< 0.001
HIV Correct knowledge and Rejection of myths						
No	Ref	–	–	Ref	–	–
Yes	0.03	0.61–1.02	0.070	0.87	0.66–1.15	0.325
Ever had sex						
Yes	Ref	–	–	Ref	–	–
No	0.34	0.28–0.43	< 0.001	0.55	0.41–0.74	< 0.001

## Discussion

This is the first trend analyses of HIV prevalence and associated factors among adolescents at a population level in South Africa. The waves of nationally representative household surveys conducted in 2008, 2012 and 2017 revealed that there was a significant change in HIV prevalence over time among adolescents aged 12–19 years, from 2008 to 2017 with an overall peak of 4.1% in 2017. The findings highlights the particularly high burden of HIV among female adolescents and those who are not in school, unemployed and orphaned.

The peak in HIV prevalence in the most recent national HIV prevalence survey suggests that there is a growing population of young people living with HIV in South Africa, and if the observed trend continue in the same trajectory, will lead to hundreds of thousands more becoming HIV-positive in the coming years. Turning the tide against HIV will require accelerated efforts and more concentrated focus to address the epidemic among adolescents [1].

In the present study HIV prevalence among those who indicated they never had sex for both 12–14 and 15–19 year olds was significantly higher in recent years compared to 2008. Notably in 2017 HIV prevalence was highest among adolescents aged 15–19 years who reported never to have had penetrative virginal or anal sex. Although there might be bias as these are self-reports of engaging in sex, other findings suggest that recent increases in adolescent HIV prevalence are more likely attributable to long-term survival of adolescents who acquired HIV through mother-to-child transmission rather than sexual activity [19]. This poses challenges for HIV prevention because of the potential for onward transmission should perinatally-infected adolescents begin unprotected sexual activity. However, HIV prevalence does not measure perinatally acquired infections directly, and therefore cannot be interpreted as a proxy for recent infections [19–21]. This re-emphasises the importance of routinely and accurately measuring HIV incidence among adolescents [19].

Current findings showed that gender disparities in the HIV burden persist with adolescent girls more likely to be living with HIV than boys of the same age. In line with current findings evidence shows that the highest population risk group remains Black African female adolescents residing in the KwaZulu-Natal province [22]. This is despite the implementation of a combination of prevention strategies for HIV, including biomedical, behavioural and structural programmes targeted specifically at adolescent girls [23, 24]. If the observed trends in HIV acquisition among adolescent females continue, achievement of the UN's goal of eliminating HIV as a public health threat by 2030 [25] will be jeopardised.

Adolescent girls remain a high priority towards ending the HIV epidemic. Prevention programmes must address gender inequalities driving excessive risk among adolescent girls by engaging men during early stages of adolescence around harmful gender norms related to HIV [26, 27]. These include harmful socio-cultural norms that lead to social vulnerability such as socio-economic disparities faced by adolescent women, gender-power dynamics and gender-based violence that influence HIV risk in this population. There is also a need to address barriers related to adolescents’ poor care-seeking behaviours through provision of youth friendly services [28].

The findings suggest that attendance at an educational institution might be protective against HIV, and this has also been reported in previous research [29]. The higher burden of HIV among adolescents not in school or unemployed presents challenges in reaching adolescents because any school-based or work-place HIV programmes and interventions, would not reach adolescents that are not found in these institutions. Elsewhere, improved school attendance was associated with decline in adolescent sexual activity and substantial declines in HIV incidence and prevalence [30]. Therefore, there is a need to strengthen the adolescent component of national HIV programmes to improve the effectiveness of the HIV response especially for out of school and unemployed youth.

In line with other studies the current findings show that adolescent orphans were more likely to be HIV positive [31, 32]. Evidence suggests that orphans, whether single orphan or double orphan are at greater risk of being HIV-positive partly due to distal risk factors such as socio-economic vulnerability, psychosocial distress, poor family functioning, and sexual abuse and partly due to proximal sexual risk factors such as number, type and concurrency of sexual partnerships [32]. Many orphans may have been infected through undiagnosed vertical transmission, which presents a challenge but also a potential entry point for HIV prevention and care continuum. The programmes designed to help orphans should also reduce high-risk sexual behaviours to prevent new infections.

## Limitations

This study has several limitations that should be noted. Participant’s self-responses may have been affected by the recall and social desirability bias. The use of face-to-face interviews to obtain self-reported information on sexual activity may have resulted in adolescents under-reporting some behaviours due to the sensitive nature of the subject. It is not clear what proportion of adolescents living with HIV were infected vertically compared with direct sexual infection. This is a major limitation for determining the cause of increased HIV prevalence in adolescents. This could be due to new infections in adolescents or due to better treatment outcomes for perinatally infected children who have been well managed and are now transitioning into adolescence. If it is the latter, then this is a positive finding that points to the effectiveness of care and treatment programms while also revealing gaps in PMTCT. However, the cross-sectional nature of the study design makes it difficult to infer causality and the study is limited to assessing factors associated with HIV prevalence among adolescents. Notwithstanding these limitations this study contributes to the body of knowledge on the factors driving the increasing trend in HIV prevalence among adolescents in South Africa. Furthermore, the use of nationally representative population-based data enables the findings of the study to be generalised to adolescents in the country.

## Conclusion

The observed increasing trend and gender disparities in HIV infection suggest a need for interventions targeting adolescents and prevention programmes aimed at addressing gender inequalities driving HIV risk among adolescent girls. Furthermore, the findings highlight the need for community-based HIV programmes focusing on the out of school and unemployed youth in addition to interventions aimed at addressing school attendance and unemployment among young people. Finally, provinces with high HIV prevalence need to be prioritised in the planning of targeted prevention interventions in order to maximize impact aimed at reducing HIV infection among adolescents.

## Data Availability

The datasets used and/or analysed during the current study are available from the corresponding author on reasonable request.
